# Membrane proteins: always an insoluble problem?

**DOI:** 10.1042/BST20160025

**Published:** 2016-06-09

**Authors:** Andrea E. Rawlings

**Affiliations:** * Department of Chemistry, The University of Sheffield, Sheffield, U.K.

**Keywords:** membrane proteins, protein engineering, protein stability, protein structure

## Abstract

Membrane proteins play crucial roles in cellular processes and are often important pharmacological drug targets. The hydrophobic properties of these proteins make full structural and functional characterization challenging because of the need to use detergents or other solubilizing agents when extracting them from their native lipid membranes. To aid membrane protein research, new methodologies are required to allow these proteins to be expressed and purified cheaply, easily, in high yield and to provide water soluble proteins for subsequent study. This mini review focuses on the relatively new area of water soluble membrane proteins and in particular two innovative approaches: the redesign of membrane proteins to yield water soluble variants and how adding solubilizing fusion proteins can help to overcome these challenges. This review also looks at naturally occurring membrane proteins, which are able to exist as stable, functional, water soluble assemblies with no alteration to their native sequence.

## Introduction

Integral membrane proteins (IMPs) exist within lipid membranes. Current estimates suggest that between 15 and 30% of open reading frames in sequenced genomes encode membrane proteins [1–3]. This protein grouping performs a range of key functions vital to the cell, such as the controlled movement of molecules, nutrients and ions across lipid bilayers, as well as participating in cell signalling and motility. Therefore, it is unsurprising that approximately 60% of drugs used today target IMPs to achieve their therapeutic action [4], and this reliance on membrane proteins as drug targets is unlikely to diminish. Understanding IMPs through structural, biochemical and biophysical interrogation is a prerequisite for new therapeutic developments in order to build up a detailed and accurate picture of how particular membrane proteins function at the molecular level [3,5].

Their ability to insert and remain stable in lipid bilayers renders IMPs, by their very nature, intrinsically hydrophobic and as such they have low solubility in aqueous environments. The poor water solubility of these proteins creates a challenge to successful *in vitro* membrane protein characterization. To circumvent this, detergents are often used to solubilize the membrane proteins [6–8]. The detergent molecules form a micelle structure, which encircles the membrane protein and provide an environment with similarities to the natural lipid surroundings. However, detergents are not without their own problems. Finding detergents and buffer conditions which provide optimal protein stability without loss of function is often a time consuming process of trial and error [7,9,10], although some high-throughput methods have been developed to aid in this search [9,10]. It is also necessary to maintain the concentration of the detergent above the critical micelle concentration (CMC) at all times to prevent the dissolution of the micelle–protein complex [7,8]. There are a number of alternative systems emerging for studying IMPs in a water soluble form, including the use of amphipols [11], bicelles [12] and nanodiscs [13], although these often require the membrane protein to be isolated in detergents prior to incorporation into the new system.

**Figure 1:**
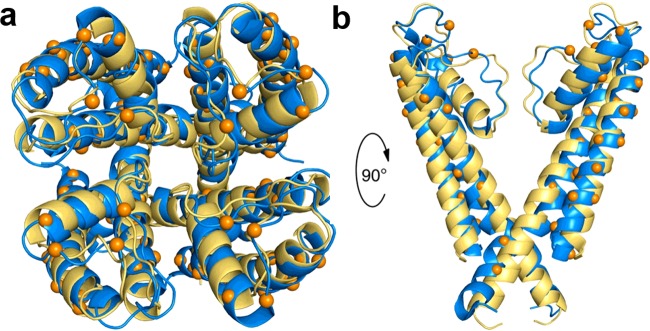
Retention of structure in water soluble KcsA Structural comparison of the transmembrane region of the native (PDB: 1K4C) [15] and water soluble variant (PDB: 2K1E) [18] of the bacterial potassium ion channel KcsA. Native KcsA in yellow and water soluble KcsA in blue. Orange spheres indicate the Cα positions of the mutated residues in the water soluble variant. Shown here are top-down four subunits (**a**) and side-on two subunit views (**b**). Figure reproduced from [14]: Perez-Aguilar, J. and Saven, J. (2012) Computational Design of Membrane Proteins. Structure **20**, 5–14.

## Water soluble membrane proteins by computational redesign

A radical strategy to overcome some of the difficulties in working with IMPs is to redesign the protein to become water soluble by substituting the exterior lipid facing hydrophobic residues of the protein for hydrophilic ones [14]. As is the case for many soluble proteins, the residues buried within the protein core are mainly responsible for generating the correct protein structure and functional activity. By leaving these interior residues of the protein unchanged, the overall structure and function of the protein is retained, but now without the need for external solubilizing agents (e.g. detergents). One of the challenges with this approach is deciding which residues to modify. The first example of the computationally redesigned multi-transmembrane spanning protein was the potassium channel KcsA. In this case, a crystal structure of the tetrameric assembly was already available [15], allowing the lipid exposed residues to be readily identified. The solvation propensities and molecular potentials of residue substitutions at these positions were then modelled computationally using a probabilistic design method to arrive at 29 transmembrane residue substitutions per subunit of the tetrameric assembly (27% of the residues in the transmembrane regions) [16,17]. When this KcsA variant was expressed in *Escherichia coli*, the protein was produced and isolated in high yields and maintained solubility without the need for detergent solubilization. Size-exclusion chromatography and analytical ultracentrifugation revealed that the protein assembled into tetrameric oligomers similar to that of the wild-type protein [16]. Analysis of the structure of the soluble variant by NMR showed that it was highly similar to that of the wild-type KcsA (Figure 1) [15,18]. This is a remarkable finding considering the high number of mutated residues and the obvious differences between lipid and water as solvents. This highlights that the short-range backbone interactions and side-chain packing drive assembly of secondary and tertiary structures in membrane proteins [19]. Not only is the structure of the water soluble KcsA variant highly similar to the wild-type protein, but it also displays NMR signal intensities which are 5-fold more sensitive for potassium than sodium ions in the region of the ion selectivity filter. This suggests that the soluble variant may retain functional relevance [18] as previous biochemical and structural studies of KcsA have recorded selectivity for potassium over sodium of 5–to 7–fold [20,21]. Similar methodologies have been successfully applied to a range of different IMPs including phospholamban, [22] the nicotinic acetylcholine receptor [23] and the human μ-opioid receptor [24,25]. In all these cases, the water soluble variants are able to bind their target receptor molecules, making them potentially useful for initial drug screening studies which are often particularly reliant on the availability of large quantities of purified, stable, membrane proteins.

## Water soluble membrane proteins through solubility enhancing fusion proteins

A limitation of redesigned, water soluble IMP's is that a large number of mutations are required. Even though the generated protein may have similar overall structural and biophysical characteristics, it is likely to feature many subtle changes which could produce alterations to the proteins function. An alternative strategy would be to keep the membrane protein sequence unchanged but to supplement the construct with fusion tags which could enhance the solubility of the IMP while maintaining the correct fold and functional form of the protein. This approach was recently realized by Mizrachi et al. [26,27]. In their method, which they term SIMPLEx (solubilization of IMPs with high levels of expression), they utilize an amphipathic protein as a fusion partner to bring about a range of water soluble membrane proteins [27].

**Figure 2:**
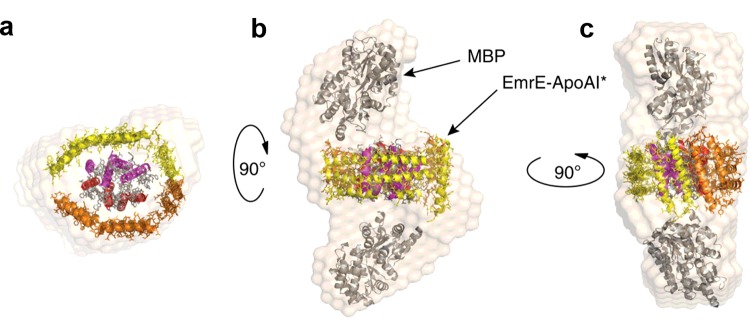
Arrangement of an apolipoprotein domain fusion tag surrounding the integral membrane protein EmrE Structural reconstruction based on SAXS analysis of ΔspMBP–EmrE–ApoAI* fusion protein [27]. (**a**) ΔspMBP has been removed from the reconstruction to allow visualization of ApoAI* (orange/yellow) shielding EmrE. (**b**–**c**) Side views of the chimaera with ΔspMBP present. Figure reproduced from [27]: Mizrachi, D., Chen, Y., Liu, J., Peng, H., Ke, A., Pollack, L., Turner, R., Auchus, R. and DeLisa, M. (2015) Making water-soluble integral membrane proteins *in vivo* using an amphipathic protein fusion strategy. Nat. Commun. **6**.

As many of the newly developed solubilization agents for IMPs are amphipathic in nature [28], the authors selected the approximately 200-residue C-terminal lipid binding domain of apolipoprotein from *E. coli* as the solubilizing fusion partner. As well as being amphipathic, this protein has a high degree of structural flexibility allowing it to easily adapt to a variety of different geometries when required [29]. This protein domain, abbreviated to ApoAI*, is already used to bind phospholipid molecules in nanodiscs, into which detergent solubilized IMPs can be partitioned, making its selection as a fusion partner a natural choice [13]. A construct encoding ApoAI* as a C-terminal fusion partner to the ethidium multidrug resistance protein E (EmrE) was created. EmrE is a relatively small α-helical IMP. In order to prevent insertion of the EmrE–ApoAI* fusion into the inner cell membrane an N-terminal decoy protein, outer surface protein A (OspA) (or MBP, maltose binding protein), was added as well. This protein chimaera was expressed in *E. coli* and subsequent Western blot analysis showed partitioning of the protein to the water soluble cell fraction. This is a remarkable result considering that when EmrE was produced alone it is present only within the cell membrane and insoluble fractions [27]. The chimaera could be successfully purified in high yield of between 10 and 15 mg/l of cell culture by conventional nickel affinity purification without the aid of any detergents. However, was the ApoAI* binding high numbers of lipid molecules as it does in nanodiscs? Lipid analysis of the purified chimaera showed only 5–10 lipid molecules per monomer of ApoAI*, rather than the expected 70, showing that the ApoAI* fusion does not solubilize the IMP by simply binding high amounts of lipid but rather by interacting directly with the IMP itself [25]. Crucially, the purified chimaera was analysed by size-exclusion chromatography and showed retention times equivalent to the presence of dimeric and tetrameric species, confirming that the presence of the fusion tag had not interfered with the normal assembly of EmrE into dimers and tetramers. The EmrE chimaera was also able to bind substrates with close to native affinity. SAXS analysis of a ΔspMBP–EmrE–ApoAI* fusion demonstrates that the ApoAI* domains assemble around the membrane protein core (Figure 2) to create a solubilizing protein shell (ΔspMBP refers to maltose-binding protein lacking the signal peptide). This ground breaking approach for *in vivo* water solubilized IMP production has now been applied to several other membrane proteins with differing size and structures and shown to give similar results [27]. These include among others outer membrane protein X (OmpX) and disulfide bond formation protein B (DsbB) from *E. coli*, cytochrome *b*_5_, Claudin-1 and 3 from *Homo sapiens* and voltage dependent anion selective channel 1 (VDAC1) from *Rattus norvegicus* [27]. This technique has the potential to revolutionize membrane protein research by increasing the ease of production and thereby significantly improving the yield of functional protein for structural and biophysical study.

## Naturally occurring water soluble membrane proteins

This review has so far focused on IMPs which normally reside within lipid membranes and which can, by elaborate protein engineering methods, be made to exist in a water soluble form without the addition of detergents or other solubilizing agents. However, this does not represent the whole picture. There are naturally occurring membrane proteins which reportedly exist in a water soluble state with little or no modification of their sequence. Many of these are found in the magnetosomes of magnetotactic bacteria (MTB). MTB are naturally occurring bacteria able to synthesize precise crystals of magnetic nanoparticles inside their cells [30,31]. These nanocrystals usually take the form of the iron oxide magnetite, and are made within an internal lipid vesicle termed the magnetosome [31]. This lipid vesicle can be considered as an organelle and is rich in various proteins which are believed to control all aspects of the nucleation, growth and maturation of the crystalline magnetite core [32–36].

**Figure 3:**
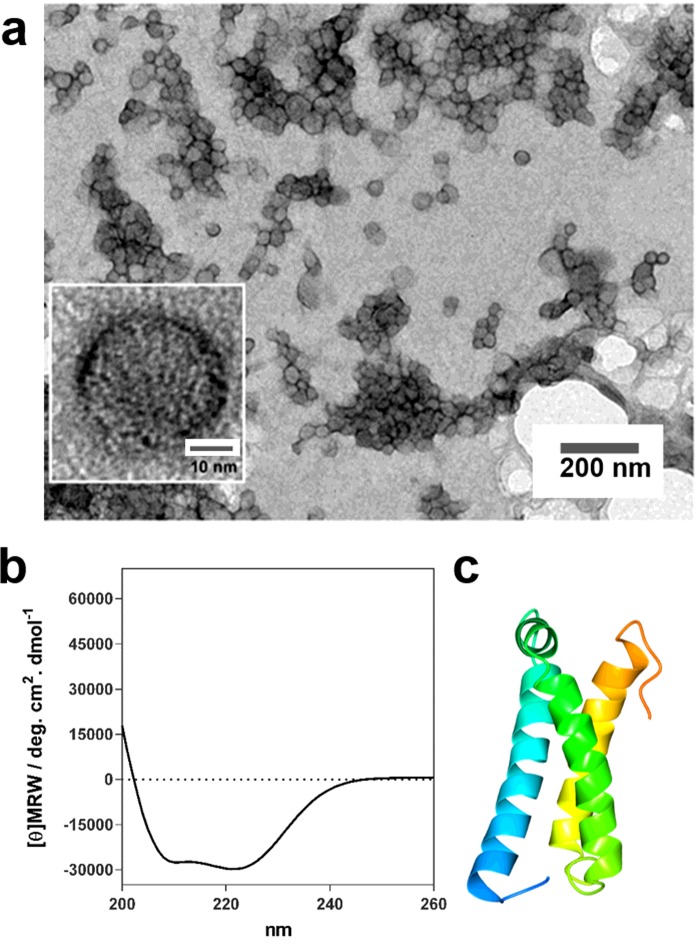
Water soluble magnetosome membrane protein MmsF MmsF soluble proteinosome structures [44]. (**a**) Negatively stained TEM images of MmsF at different magnifications. Scale bar of inset is 10 nm. (**b**) CD analysis of MmsF showing α-helical secondary structure. (**c**) Structure prediction of MmsF from the Quark server [49] showing three helices coloured blue to orange from N- to C-termini. Figure reproduced from [44]: Rawlings, A.E., Bramble, J.P., Walker, R., Bain, J., Galloway, J.M. and Staniland, S.S. (2014) Self-assembled MmsF proteinosomes control magnetite nanoparticle formation *in vitro.* Proc. Natl. Acad. Sci. U.S.A. **111**, 19094–19099.

The first of these proteins to be isolated and studied is the magnetosome membrane specific protein 6 (Mms6) [37]. This is a small, 6 kDa protein with a hydrophobic N-terminal domain and hydrophilic, acid-rich, C-terminal domain. Mms6 normally resides in the magnetosome membrane but has been expressed successfully in *E. coli* where it is found to form insoluble inclusion bodies [37]. These inclusion bodies can be dissolved in high concentrations of guanidine or urea and refolded using various refolding strategies to reduce the concentration of the denaturant. Remarkably, after removal of the denaturant, the Mms6 protein is folded, water soluble and forms micellar structures with the N-terminal regions buried and the C-terminal regions surface accessible [37]. These water soluble Mms6 micelles are able to bind to iron ions with high affinity [37,38] via the negatively-charged carboxylic acid groups present in its hydrophilic region, and in so doing transform their structure into a planar disc-like assembly [39]. This iron binding ability is thought to be important for magnetite formation within the magnetosome. Purified Mms6 has also been shown to incorporate into liposomes where the N-terminal hydrophobic region becomes resistant to proteolytic cleavage by proteinase K but the C-terminal region retains solvent (and therefore protease) accessibility [38]. Due to its small size, Mms6 can also be produced as a water soluble fusion protein to the large solubility enhancing tag MBP, and then released via the action of the sequence selective tobacco etch virus (TEV) protease [40]. In that case, there is no need for refolding and the protein shows similar properties to refolded Mms6. Perhaps the most intriguing aspect of Mms6 is the ability of the purified micelles to influence the characteristics of magnetite nanoparticles when included as an additive in synthetic magnetite precipitation reactions. The particles formed in the presence of Mms6 display a narrower range of sizes, mineral types and often have the cubo-octahedral appearance of biogenic magnetosome derived nanoparticles [37,41–43] compared with particles produced without protein. This activity indicates that the water soluble micellar form of Mms6, retains a function which is similar to its proposed role *in vivo*.

Four other proteins, magnetosome membrane specific protein F (MmsF), magnetosome associated membrane protein F (MamF) and magnetosome membrane unknown protein F (MmxF), from *Magnetospirillum magneticum* AMB-1 [44] and MamC from *Magnetococcus marinus* MC-1 [45,46], have also been found to be water soluble. Transmembrane prediction algorithms suggest that the three highly similar proteins from *M. magneticum* AMB-1 possess three transmembrane helices [44,47] and *in vivo* GFP localization studies of MmsF confirm that this protein is found at the magnetosome membrane [48]. However, when overexpressed in *E. coli*, MmsF, MamF and MmxF were found only in the water soluble cell fraction and not in the cell membrane or in inclusion bodies as would be expected for a typical membrane protein [44]. Following affinity purification the proteins were analysed by dynamic light scattering, TEM and CD, and were found to be assembled into large spherical structures resembling vesicles and to have a significant α-helical content consistent with them being α-helical polytopic membrane proteins [44] (Figure 3). The vesicle structures or proteinosomes, were approximately 40 nm in diameter and were sensitive to proteolysis with proteinase K. The authors found that when added to a synthetic magnetite precipitation reaction, these proteins could influence the balance of the various iron oxides which formed [44].

MamC has two predicted membrane spanning helices and is a protein unique to the magnetosome membrane [46,47]. Overexpression in *E. coli* gives rise to inclusion bodies of MamC which can be refolded after dissolution in urea to generate water soluble protein micelles [45]. The soluble form of this protein is able bind iron ions [46] and also to influence the size of magnetite nanoparticles formed during synthetic reactions [45].

Several of the membrane proteins from the magnetosomes of MTB appear able to exist both within the magnetosome membrane and as water soluble micelles and assemblies. The common hallmark between all of these proteins appears to be their ability to achieve aqueous solubility through aggregation and shielding of their hydrophobic transmembrane spanning regions. This is coupled with highly charged solvent exposed regions containing large numbers of acidic amino acids. Why do these proteins have this dual ability? This is a question that remains to be answered. However, to find several proteins with this ability which all normally reside together in the same membrane is, I believe, unlikely to be a serendipitous occurrence. It is possible these proteins form water soluble assemblies before they are recruited and inserted into the magnetosome membrane to prevent their incorporation into the inner cell membrane. Unlocking the specific sequence and structural motifs, as well as discovering new proteins with similar properties will no doubt guide the development of new approaches to the design of water soluble membrane proteins.

## Conclusions

Our general understanding of proteins tells us that they will normally be a member of one or two different, and mutually incompatible classifications: either soluble or membrane associated. This mini review has shown that some proteins can actually be both whereas others can be converted from one to the other through rational design. Membrane proteins which are water soluble bring many of the advantages of typical soluble proteins such as high yield from overexpression, ease of purification and stability during biophysical investigations [16,18,23,27,44,45]. Unfortunately these approaches do come with their own limitations. For instance, using the highly flexible apolipoprotein domains as solubilizing fusion proteins may hamper efforts to crystallize IMPs [27], and making large numbers of mutations in the sequences of membrane proteins to render them water soluble could cause unanticipated functional changes, altering the very function the engineered protein was designed to test. However, these difficulties are not insurmountable, and with each new membrane protein that is investigated in a water soluble form comes new knowledge of fundamental membrane protein biology to lead the development of future advances in this vital research area.

## Abbreviations

EmrE: ethidium multidrug resistance protein E
IMP: integral membrane protein
MamF/C: magnetosome associated membrane protein F/C
MBP: maltose binding protein
Mms6: magnetosome membrane specific protein 6
MmsF: magnetosome membrane specific protein F
MmxF: magnetosome membrane unknown protein F
MTB: magnetotactic bacteria
PDB: protein data bank
